# Strengthening of CoNiFeV_0.5_Mo_0.2_ Medium Entropy Alloy Wire Rods with Loading-Unloading Cycles

**DOI:** 10.3390/ma15041493

**Published:** 2022-02-17

**Authors:** Xulong An, Chenglin Chu, Wenwen Sun, Wei Wei

**Affiliations:** 1 School of Materials Science and Engineering, Changzhou University, Changzhou 213164, China; benjamin.wwei@163.com; 2 School of Materials Science and Engineering and Jiangsu Key Laboratory for Advanced Metallic Materials, Southeast University, Nanjing 211189, China; swwcsu@live.cn

**Keywords:** CoNiFeV_0.5_Mo_0.2_, medium entropy alloy (MEA), loading-unloading cycles, textures, mechanical properties

## Abstract

Changes in the texture as well as mechanical properties of CoNiFeV_0.5_Mo_0.2_ medium entropy alloy wire rods during loading–unloading are investigated. The intensity of the recrystallization texture {001}<110> component and fraction of low angle grains increase with the loading–unloading cycles and the alloy strength increases (934 MPa to 1083 MPa) due to dislocation increment in the loading–unloading cycles. The loading modulus (E_l_) and average modulus (E_secant_) for a hysteresis loop decrease slightly, whereas the unloading modulus (E_un_) increases, the Eun increment of 5-TC-UTand 10-TC-UT are 22 and 137 GPa.

## 1. Introduction

In modern engineering systems, materials which can withstand extreme working and environmental conditions are in high demand. Recently, multi-principal-element alloys (MPEAs) have been extensively studied due to their promising mechanical properties [1,2,3,4,5]. Based on the configurational entropy, alloys are classified to have low entropy (ΔS_conf_ < 1 R), medium entropy (1 R ≤ ΔS_conf_ ≤ 1.5 R), and high entropy (ΔS_conf_ > 1.5 R), where R is the gas constant, 8.314 J/K mol. Most traditional alloys have low entropy, but the majority of multi-principal-element alloys have medium or high entropy [6,7]. To optimize the properties of MPEAs, several methods have been proposed, for example, transformation induced plasticity [8,9], fine grain strengthening [10,11], second phase strengthening [12,13], solid solution strengthening [14,15], and concentration wave regulation strengthening [16].

There have been investigations to understand the microstructure and properties of as-cast HEAs [17,18,19]. Casting is still the main forming technology for HEAs and the as-cast ingots typically have large grain sizes, significant porosity, and coarse second phase particles. The casting defects of HEAs such as the casting porosity, composition, segregation, etc., during solidification impact practical implementation. Thermo-mechanical processing such as forging, rolling, and drawing can improve the alloy properties by refining the microstructure and reducing/eliminating defects. From the commercial (economic) point of view, rolling is the most important metal working and shaping technique and is used to manufacture products such as sheets, beams, rails, and wire rods. Besides, the amount of elastic recovery, more often called springback, is determined by the elastic–plastic properties of the sheet material, tool design and the forming process. The experimental observations have shown that metallic materials, including steels and aluminum alloys, show a significant departure from linearity, such as a hysteresis loop during the unloading and reloading [20]. However, there is few studies on the modulus changes of loading and unloading in HEAs. To understand comprehensively the properties of HEAs, it is necessary to take precise loading-unloading behavior in consideration.

In this study, CoNiFeV_0.5_Mo_0.2_ medium entropy alloy (MEA) wire rods with a diameters of 1.5 mm are prepared by casting, forging, and cold rolling and the changes in the textures and mechanical properties during loading–unloading are investigated systematically.

## 2. Materials and Methods

**Figure 1:**
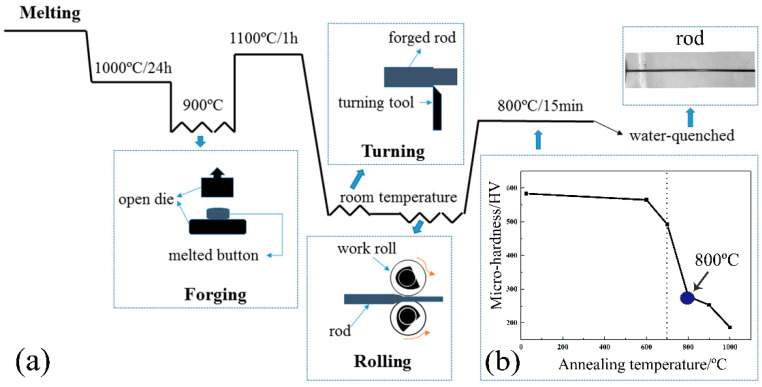
(**a**) Schematics of thermos-mechanical processing of specimens: forging, turning and rolling; (**b**) Micro-hardness of specimens at different annealing temperatures.

The CoNiFeV_0.5_Mo_0.2_ MEA was prepared by arc-melting of pure Fe, Co, Ni, V and Mo metals (>99.9 purity), as described in Figure 1a. The arc-melted buttons were re-melted and flipped under electromagnetic stirring five times to ensure good homogeneity and then homogenized in vacuum at 1000 °C for 24 h before hot forging into 10 mm diameter rods at 900 °C. The as-forged rods were annealed at 1100 °C for 1 h. The wire rods 1.5 mm in diameter were obtained by turning and multi-step cold-rolling while the diameter was reduced by 80%. According to the micro-hardness curve of the specimens annealed at different temperature (Figure 1b), the rolled rod fully recrystallized at above 700 °C. To optimize the microstructure and workability, the as-rolled rods were annealed at 800 °C for 15 min and then water-quenched.

The microstructure of the specimens was characterized on a scanning electron microscope (SEM) equipped with an energy-dispersive X-ray spectrometer (EDS) and a transmission electron microscope (TEM). The specimens were ground with 500~5000 grid sandpapers and scratches were removed from the EBSD specimens by electrolytic polishing in a solution containing 10 vol% perchloric acid and 90% acetic acid. The deformation textures and grain distributions were studied by electron backscattering diffraction (EBSD) with a step size of 0.5 μm, and the data was analyzed by the HKL CHANNEL5 software.

A CMT 5105 universal electronic tensile testing machine was used for monotonic and cyclic loading–unloading at a strain rate of 1 × 10^−3^ s^−1^ and the elongation was measured with an electronic extensometer. Rod-like tensile specimens with a gauge length of 10 mm and diameter of 1.5 mm were used in the tensile experiments.

Every unloading–loading process produced a hysteresis loop. Five loading–unloading cycles produced five hysteresis loops with a 2% strain increment and 10 loading–unloading cycles with a 4% strain increment produced ten hysteresis loops. The as-rolled CoNiFeV_0.5_Mo_0.2_ MEA rods were subjected to different numbers of loading–unloading cycles, namely 0, 5, and 10 (samples denoted as UT, 5-TC-UT, 10-TC-UT, respectively).

## 3. Results and Discussion

**Figure 2:**
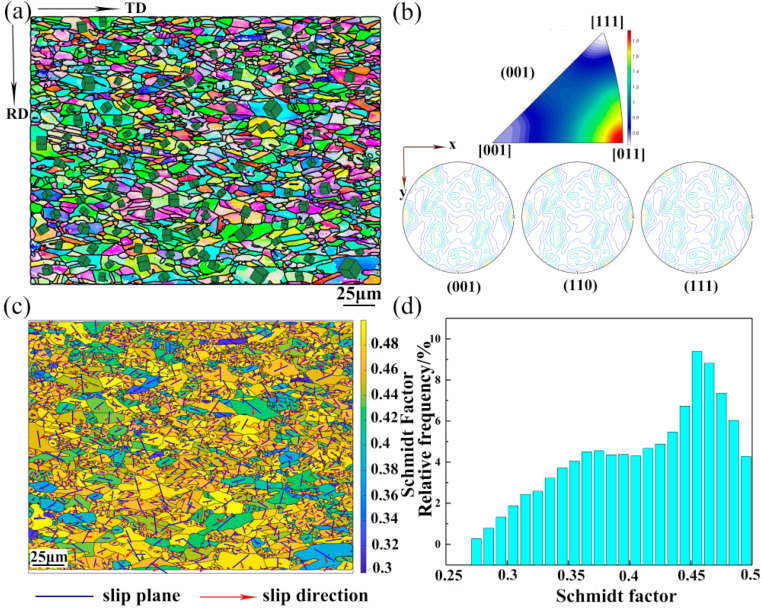
(**a**) EBSD IPF image of the UT specimen with the crystal orientations of the big grains (the average grain size is about 20 μm) shown by the crystal shape orientated according to the orientation of the grains; (**b**) Grain orientation of each grain in the (001) plane and (001), (110), (111) pole figures of (**a**); (**c**) Schmidt factors calculated by the slip systems as well as stress tensor in the crystal coordinates; and (**d**) Schmidt factor distributions (histogram) of the UT specimen.

EBSD was performed to investigate the crystal structure and grain orientation in the as-annealed CoNiFeV_0.5_Mo_0.2_ MEA specimens. The inverse pole figure (IPF) maps and corresponding misorientaion profiles of the UT specimen are shown Figure 2. The IPF maps show that the UT specimen consists of a fully recrystallized microstructure and no deformed microstructure can be observed (Figure 2a). The (001), (001), and (001) PF (Figure 2b) indicate a strong texture in the UT specimens. The recrystallization texture component {001}<110> is observed from the (001) IPF of the UT specimens.

Activation of twins and slip systems usually obey the rule of Schmidt during plastic deformation of metals. The Schmidt factor can be calculated as follows:

(1) $$m_{s}=\cos\varphi \cdot \cos\lambda$$

where *m_s_* represents the Schmidt factor, φ and λ represent the angle between the normal axis of the force axis and sliding surface as well as the angle between the normal axis of the force axis and sliding direction, respectively. Plastic deformation of metals occurs by slipping and a larger Schmidt factor implies a larger probability for the slip system to start. Figure 2c shows the Schmidt factor distribution maps of each slipping system. The Schmidt factor is between 0.3 and 0.5 and the slip systems of the UT specimen are presented in Figure 2d. It can be observed that the slip system has a higher frequency between 0.38 and 0.45. The appearance and evolution of the texture mainly depends on the movement of the slip system and twin system and therefore, the start of the slip system with the Schmidt factors between 0.38 and 0.45 leads to the formation of the {001}<011> texture in the UT specimens.

**Figure 3:**
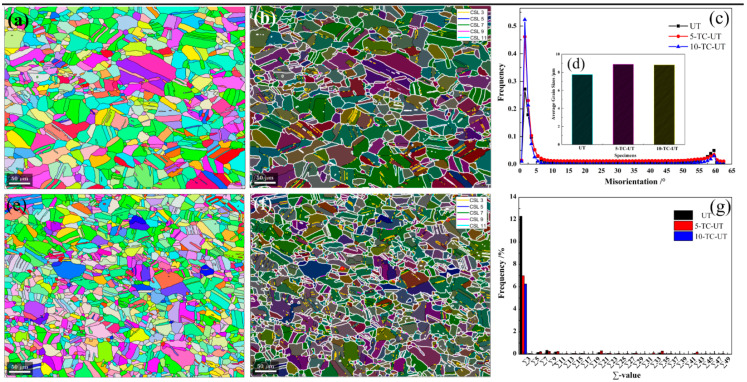
(**a**,**e**) EBSD IPF images of the 5-TC-UT and 10-TC-UT specimens; (**b**,**f**) CSL boundaries of the 5-TC-UT and 10-TC-UT specimens; (**c**,**d**) grain misorientation distribution and grain size of the UT, 5-TC-UT and 10-TC-UT specimens; (**g**) ∑ boundaries distribution histogram of the UT, 5-TC-UT and 10-TC-UT specimens.

Figure 3 shows the EBSD results of the 5-TC-UT and 10-TC-UT specimens and annealing twins are observed in both specimens. The average grain size of the 5-TC-UT and 10-TC-UT specimens is similar and about 9 μm, which is bigger than that of the UT specimen (Figure 3d). The misorientation angle distribution curves of the UT, 5-TC-UT and 10-TC-UT specimens (Figure 3c) show that the misorientation angle distributions have a strong preference of 1.5°, indicating that the subgrain boundary has the highest fraction in the grain boundaries of the as-rolled CoNiFeV_0.5_Mo_0.2_ MEA. The fraction of low angle grain boundaries (LAGBs) (misorientation angle < 10°) is beyond that of the high angle grain boundaries (HAGBs) (misorientation angle > 10°). The fraction of LAGBs increases with loading–unloading cycles in the tensile test and the proportion of LAGBs in the 10-TC-UT specimen is larger than that in the UT and 5-TC-UT specimens.

To investigate the relationship between the mechanical properties and characteristic grain boundary distributions, the main coincidence site lattice (CSL) boundary maps of the 5-TC-UT and 10-TC-UT specimens are plotted in Figure 3b,f. The LAGBs/HAGBs, Σ3, Σ5, Σ7, and Σ9 boundaries are displayed in white, yellow, blue, red, and pink, respectively. It is obvious that the LAGBs/HAGBs proportion is higher than that of CSL boundaries. The CSL boundary distribution curves of the UT, 5-TC-UT, and 10-TC-UT specimens are shown in Figure 3g. The fraction of the Σ3 boundary in CSL boundaries is the largest and the fraction of Σ3 boundaries in total CSL boundaries increase with decreasing number of loading–unloading cycles in the tensile test.

**Figure 4:**
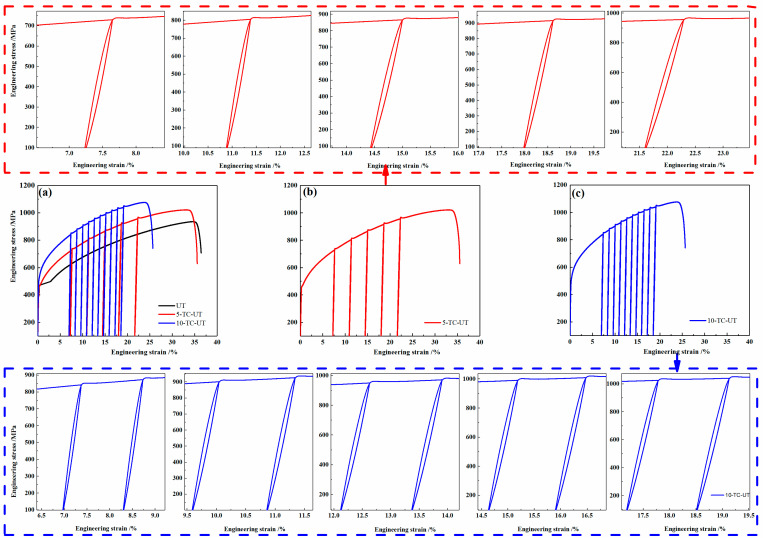
(**a**) Engineering stress–strain curves of the UT, 5-TC-UT, and 10-TC-UT specimens; (**b**,**c**) engineering stress–strain curves of the 5-TC-UT specimen with intermittent hysteresis loops for every 2% strain increment and 10-TC-UT specimens with intermittent hysteresis loops for every 4% increment. Each hysteresis loop is enlarged.

Based on Hooke’s law, the deformation materials completely recover during unloading in the elastic stage. The loading and unloading moduli are the same and the area enclosed by the hysteresis loop is zero, which are opposite to those in the plastic stage. Therefore, it is meaningful to investigate the influence of the loading–unloading cycles on the mechanical properties during plastic deformation. Figure 4a presents the monotonic and cyclic engineering stress–strain curves of the CoNiFeV_0.5_Mo_0.2_ MEA. The stress–stain curves of the three specimens exhibit approximate the same yield strength of about 460 MPa, indicating that cyclic loading–unloading during plastic deformation has no effect on the yield strength of the as-rolled CoNiFeV_0.5_Mo_0.2_ MEA. The ultimate strength and elongation of the UT, 5-TC-UT, and 10-TC-UT specimens are 998 MPa (35%), 1022 MPa (34%), and 1072 MPa (24%), respectively. It is obvious that cyclic loading–unloading can improve the strength but weaken the ductility of the alloy. Figure 4b,c show the engineering stress–strain curves with five intermittent hysteresis loops for every 2% strain for the 5-TC-UT specimen and with ten intermittent hysteresis loops for every 4% strain for the 10-TC-UT specimen, clearly demonstrating the nonlinear loading and unloading response. The slopes of both the loading and unloading curves decrease from the beginning of loading and unloading.

**Figure 5:**
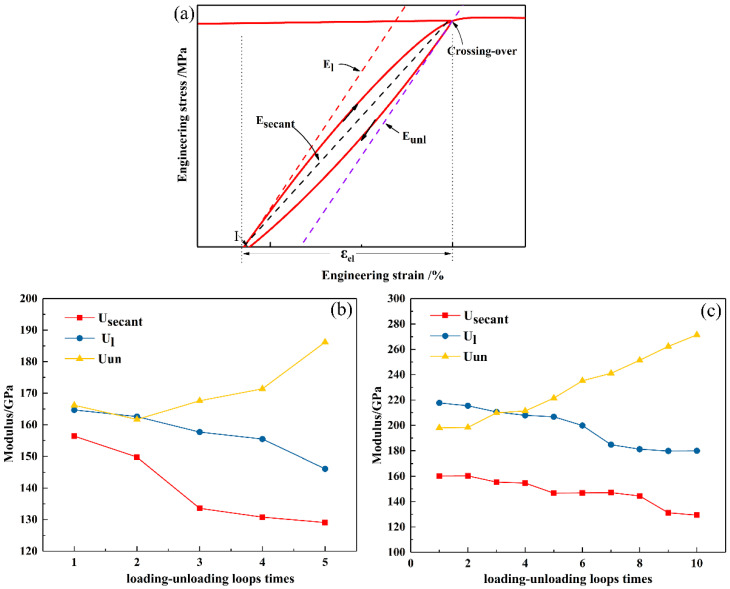
(**a**) Schematic engineering stress–strain curve from the cyclic loading–unloading tensile tests; (**b**) secant modulus E_secant_, loading modulus E_l_, and unloading modulus E_un_ of 5-TC-UT; (**c**) secant modulus E_secant_, loading modulus E_l_, and unloading modulus E_un_ of 10-TC-UT.

Owing to higher stress levels during loading than unloading, a hysteresis loop is formed in a loading–unloading tensile cycle. Figure 5a illustrates the loading–unloading hysteresis loop, where E_l_ and E_un_ represent the loading modulus and unloading modulus, respectively. They are calculated from the slope of the ascending branch and descending branch in the hysteresis loop. The average modulus of a hysteresis loop is denoted by the secant modulus (E_secant_).

Cyclic loading–unloading can increase residual stress, generate micro-cracks and variate the dislocation structure [21]. The residual stress, which is normally the amount of stress variations raised in the microstructure as a result of the cyclic processing, becomes more severe with deformation and can retard the elastic recovery, thus reduced the elastic modulus. Another factor that may change with the plastic deformation is the dislocation density in the microstructure. The pile-up dislocations during the loading can move in the backward direction during the unloading, as the shear stress acting on the slip system is released. The dislocations in the pile-up cause deformation during unloading, and they can also induce deformation during reloading when the applied stress is smaller than the yield stress.

Dislocations formed during plastic deformation are often repulsive to each other, and they are normally forced to get closer only by the applied stress. As the applied stress drops, the dislocations can restore their previous equilibrium positions with associated strain and reduced elastic modulus. The amount of the decrease in E_scant_ and E_l_ is associated with the dislocation density increment, while the increment in E_un_ is the result of the backward motion of dislocations [20].

To investigate the influence of the hysteresis loops on the mechanical properties of alloy, E_l_, E_un_, and E_secant_ are extracted from the engineering stress–strain curves of the UT, 5-TC-UT, and 10-TC-UT specimens (Figure 5b,c). For the 5-TC-UT specimen, the loading–unloading cycles decrease E_l_ and E_secant_ slightly, but increase E_un_ by 166 GPa from 188 GPa. The moduli exhibit a different behavior in the 10-TC-UT specimen. The average elastic modulus E_secant_ shows a small change in a narrow range from 140 GPa to 160 GPa. The loading modulus E_l_ decreases slightly, but the unloading modulus E_un_ increases obviously from 197 GPa to 370 GPa.

**Figure 6:**
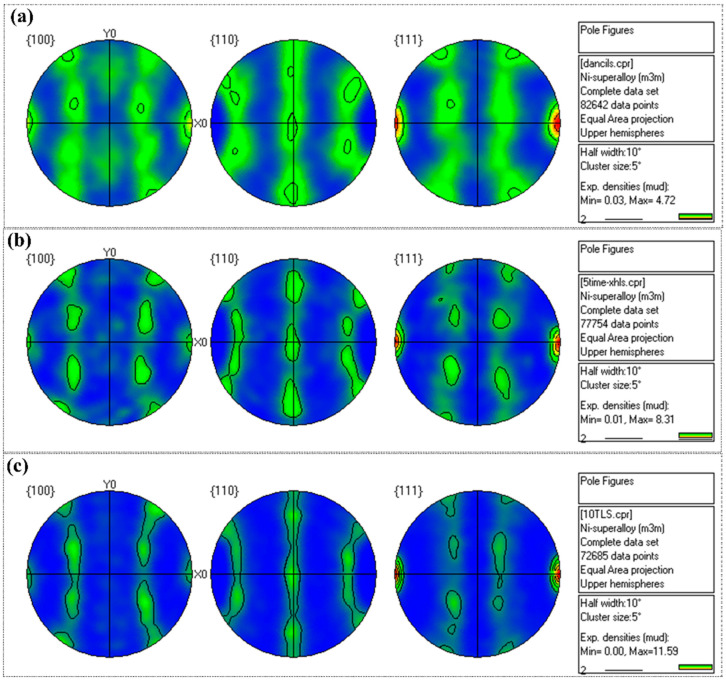
Pole figure from the EBSD data showing the texture intensity (**a**) Pole figure of UT, (**b**) 5-TC-UT, and (**c**) 10-TC-UT specimens.

The as-rolled CoNiFeV_0.5_Mo_0.2_ MEA has a strong texture. Preferential growth of the texture components involves rapid migration of boundaries, which in turn depends on rapid diffusivity of atoms across the boundaries [22]. The recrystallization texture depends on the deformed microstructure (e.g., deformed band) [23]. The number of loading-unloading cycles has an obvious effect on the intensity of the texture and a reasonable explanation is that the loading–unloading cycles lead to transformation from the initial deformation texture to the {001}<011> texture. As shown in Figure 6, the texture intensity increases with loading-unloading cycles. The texture intensity of the 10-TC-UT specimen is 11.59 mud and bigger than those of UT (4.72 mud) and 5-TC-UT (8.31 mud). Besides, the texture evolution during plastic deformation affects the elastic modulus [20].

The evolution of the tensile strength of the as-rolled CoNiFeV_0.5_Mo_0.2_ MEA for different loading–unloading cycles shows a close relationship with dislocations. The mobile dislocation density increases during loading because of activation of dislocations and decreases during unloading due to annihilation and runback. During loading, the intersections of dislocations on many slip planes increase the number of immobile tangles pile-ups and forests [24]. During unloading, the intertwined and piled up dislocations become loose and begin to move in the reverse direction regarded to be the elastic bank flow [25]. Grain boundaries are interfacial defects that bind together crystallites of various shape, size, and spatial orientations within the polycrystalline materials. During plastic deformation, the released dislocations move along slip planes and easily pile up when stopped. The movable pile-up dislocations can relax or move backward slightly when the applied stress is released during unloading, loading to a small amount of non-elastic deformation, or so-called micro-plasticity [26]. This micro-plastic strain also increases with loading–unloading cycles to improve the alloy strength. A low-angle grain boundary (LAGB) can be described as an array of lattice dislocations [27] and in the CoNiFeV_0.5_Mo_0.2_ MEA, the fraction of LAGBs increases with loading–unloading cycles (Figure 2c) resulting from occurrence and propagation of dislocations.

**Figure 7:**
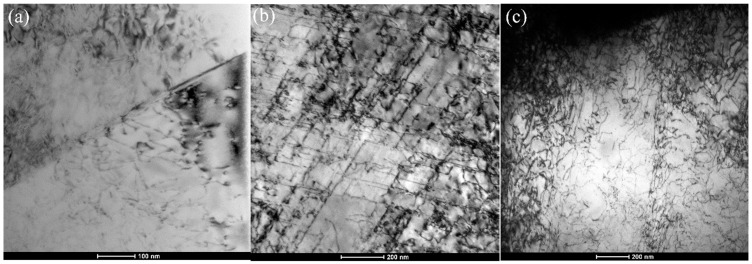
The dislocations in (**a**) UT, (**b**) 5-TC-UT, and (**c**) 10-TC-UT specimens.

Figure 7 illustrates the variations of dislocations in the specimens after monotonic and cyclic loading–unloading. The moving dislocations are nonparallel to other dislocations and they can intersect each other if the external stress is high enough to overcome the elastic interaction force. The internal stress is stored during cyclic loading–unloading and provides a new driving force to the dislocation intersection process. With increasing strain during tensile deformation, dislocation arrangement changes from isolated dislocations to a dislocation forest structure [28]. The dislocation structure becomes heterogeneous with a region of low dislocation density and others with a high density. The dislocation structure constitutes the LAGBs which become new obstacles to hinder the dislocation motion. It is thus reasonable that the ultimate strength of the CoNiFeV_0.5_Mo_0.2_ MEA increases with loading–unloading cycles (Figure 4a).

## 4. Conclusions

In this study, the influence of the number of loading–unloading cycles on the evolution of the texture and mechanical properties is investigated. The recrystallization texture component {001}<110> is observed and the intensity increases with loading–unloading cycles. With increasing loading–unloading cycles, the fraction of LAGBs and tensile strength increase, while the ductility weakens. Cyclic loading–unloading decreases the loading modulus (E_l_) slightly and average modulus for a hysteresis loop (E_secant_), whereas the unloading modulus (E_un_) increases. The larger low-angle grain boundary fraction resulting from more dislocations improves the strength of the as-rolled CoNiFeV_0.5_Mo_0.2_ MEA.
